# Supplementary material for: Biochemical response of Sonneratia alba Sm. branches infested by a wood boring moth (Gazi Bay, Kenya)
Source: PLoS One. 2021 Nov 2;16(11):e0259261. doi: 10.1371/journal.pone.0259261 (PMC8562821; doi:10.1371/journal.pone.0259261)
Supplement: Attachment — Submitted filename: Response to Reviewers.docx [file pone.0259261.s002.docx]

Dear Editor,

I wish to resubmit our manuscript titled biochemical response of *Sonneratia alba* Sm. branches infested by a wood boring moth (Gazi Bay, Kenya). We have considered all the required changes suggested by the two reviews as much as it is possible. Below is the list the reviewers asked (in blue fonts) and our reaction towards the raised concerns (in Red fonts).

A bout Fig. 1 and Fig. 2, The map layer has been well referenced to Kenya Marine and Fisheries Research Institute (KMFRI). This is a governmental research institute where I am an employee. Being an employee I have full rights to use the map layers as long as I acknowledge it on the map. Which I have done.

AS for Fig. 2, I wish to say that this is my figure and thus no permission is needed for me to use it.

Reviewer Comments

**Reviewer #1:**

Sonneratia alba (S. alba) is an important mangrove plant grown in low intertidal zones of downstream estuarine systems of East Africa, Southeast Asia, northern Australia, Borneo and Pacific Islands, etc. The optimal growth of this plant reaches 5 to 50% seawater, indicating its capacity to tolerate high salinity and hypoxia. In this way, S. alba is ecologically significantly important. The infestation by a lepidopteran moth wood borer species is causing mortality of S. alba and create a huge ecological and economic loss. In this aspect, the topic of the present manuscript is very important and is a need of the current situation. Hence, I think the authors bring up some important issues that will spark considerable debate.
****However, the manuscript needs some changes and justification, which should be addressed to improve the quality of the paper. I do recommend this manuscript to be published in "Plos One" with major revision and the author/s need to address below comments/suggestions:

**1.** The work of the present manuscript is focused on the mangrove tree Sonneratia alba, having huge ecological importance. At the beginning of the introduction, the authors mentioned the name of the plant; however, the name of the family is missing. Here, authors need to mention it at an appropriate place because with the plant's name, giving its family name is essential.

Response: This has been done (see line 43)

**2.** In the section “Sampling sites and samples,” the authors collected samples from different ages of plants. In certain groups (i.e., A and C), the age of the plants is mentioned, while in the group B and D age of the plants is not given. Here, the authors need to mention the age of all plants.

Response: It is true that the age of the trees at some of the plots have been given in the materials and methods section whereas other plots (B and D) have not been given. The reason for this is actually given in the materials and method section where the description of the sampling plots was given. Plot B and D are natural forest that have been since time immemorial whereas plot A and C are plantation. Thus information about these two plots is available. On the other hand, in this research, as indicated in the manuscript, all efforts were done to ensure the samples were taken from the same canopy height and the branches were of similar size as much as possible.

Also it is difficult to give the general age of mangrove trees using readily available tools in plant science. This is because methods of age estimation in mangrove have not been fully developed as is the case in other trees in terrestrial forest. It is our belief that even providing the DBH does not add any scientific relevance since a fully grown mangrove may stagnant at some stage.

**3.** In the material and method section, the author did not mention the time (month and year) of the sampling. The authors need to mention it.

Response: This has been done as requested (see lines 115-116)

4. It is a well-established fact that in plants, the lignin content varies with the age of the plants. In this manuscript, authors mentioned that lignin content plays a significant role in recovery and damaging from infestation. Hence, authors need to justify the results as they collected samples from different age plants.

Response: As much as the samples were collected from trees of various ages, the number of cumulative samples was made large enough and equal between the three categories i.e. (Dead, recovered and control). To ensure lack of bias, it has been mentioned in the manuscript that all effort was put to ensure that sampled branches were taken from same canopy position and of similar size in every sampled tree. Thus the sampled branches were assumed to be of similar physiological and developmental processes in each sampled tree. The concluded results were therefore out of the influence of age and size due to the replication effect.

5. In Fig. 3, i.e., FTIR spectrum, the authors need to mention the functional groups corresponding to each of the FTIR ranges.

Response: This suggestion has been considered. See new version of Fig 3.

6. The language of the manuscript is poor, and lots of grammatical and typo errors have been seen throughout the manuscript. Authors need to check and reframe the sentences critically.

Response: We have thoroughly revised the manuscript and made many corrections, as can be observed in the *Changes marked* version of the revised manuscript.

**Reviewer #2:**

The article entitled "Biochemical response of Sonneratia alba Sm. branches infested by a wood boring moth (Gazi Bay, Kenya)" should be revised well upon the follwoing:

1- English Editing should be performed

Response: We have thoroughly revised the manuscript and made many corrections, as can be observed in the *Changes marked* version of the revised manuscript.

2- Statistical analysis should be more clear that represents the data collected

Response: this comment from the reviewer seems not clear from our opinion. However, we would like to mention that the statistical methods applied in this paper (PCA and LDA) are very commonly used methods for such research work. Furthermore, the coherence in the results is reflected by the fact that the results of PCA and LDA support each other. In addition, we applied the One-way ANOVA test to assess the statistical significance of the results.

3- Refrances should be arranged as PLOS ONE style

Response: This suggestion has also been considered according to the journal requirements.

We re-submit this work after carefully considering all the comments of the reviewers and having done the necessary changes. We once again submit that this work has not been prior submitted to any other journal for publication consideration.

On behalf of all authors,

*Elisha Mrabu Jenoh

Kenya Marine and Fisheries Research Institute

P. O. Box 81651 (80100)

Mombasa Kenya

E-mail: [elishamrabu@gmail.com](mailto:elishamrabu@gmail.com) or [emrabu@kmfri.co.ke](mailto:emrabu@kmfri.co.ke)
